# Role of biomarkers in the diagnosis of invasive aspergillosis in immunocompromised patients

**DOI:** 10.1186/s12941-022-00539-x

**Published:** 2022-11-01

**Authors:** Parisa Badiee, Fatemeh Ghasemi, Hadis Jafarian

**Affiliations:** Clinical Microbiology Research Center, Shiraz University of Medical Sciences, Shiraz, Iran

**Keywords:** Invasive aspergillosis, Procalcitonin, sTREM-1, C-reactive protein, White blood cell, Erythrocyte sedimentation rate

## Abstract

**Background:**

Invasive aspergillosis is one of the important causes of infection in immunocompromised patients. This study aimed to evaluate the roles of biomarkers in the diagnosis of invasive aspergillosis and their relationship with antifungal stewardship programs.

**Methods:**

190 sera from 52 immunocompromised patients and volunteer individuals were included in this study. 18 immunocompromised volunteers without IA and 34 patients with probable and proven aspergillosis according to the European Organization for Research and Treatment of Cancer and the Mycoses Study Group consensus definitions were entered in this study. The respective sera were evaluated for procalcitonin, soluble triggering receptor expressed on myeloid cells-1 (sTREM-1) levels; white blood cells count (WBC) count, C reactive protein (CRP), lactate dehydrogenase (LDH), and erythrocyte sedimentation rate (ESR) values. Demographic data and clinical characteristics of patients were extracted from their files.

**Results:**

The male-to-female ratio and mean age of patients were 22/12 and 38.9 years, respectively. The hematologic disorder was the most predisposing factor (29/34, 85.3%). Sensitivity of biomarkers for diagnosis of invasive aspergillosis was 70.6% (cut off value > 190 pg/mL for sTREM-1, 71% (cut off value > 260 pg/mL) for PCT, 85.3% (cut off value > 193 U/L) for LDH, 94.1% (cut off value > 8 mg/l) for CRP, 64.7% (cut off value < 5200 cells/ml) for WBC, and 85.3% (cut off value > 23 mm/h) for ESR. Twelve patients died, with significantly increased sTREM-1 levels and decreased WBC count in them.

**Conclusion:**

According to our data, evaluation of the biomarkers can help in the diagnosis, management, and prediction of the severity of *Aspergillus* infection, and the rational use of antifungal agents in immunocompromised patients.

## Background

Invasive aspergillosis (IA) is one of the dominant causes of morbidity and mortality in immunocompromised patients [1]. Infection may be associated with many factors like age, graft-versus-host disease, prolonged neutropenia, diabetes, cytomegalovirus infection, post-operative infection, renal or liver dysfunction, steroid therapy, and chemotherapy [2–4]. Preliminary diagnosis and treatment play an important role in the handling of high-risk patients. Optimizing the usage of antifungal agents (antifungal stewardship programs) has an important character in decreasing antifungal toxicity, antifungal resistance, and cost. Conventional diagnostic methods based on culture and histology of sterile clinical samples remain the cornerstone of diagnosis [5]. However, the performance of such methods in immunocompromised patients may be underlying co-morbidities [5]. Non-culture-based methods such as 1,3-b-D glucan, galactomannan, and DNA assay are reliable ones for early diagnosis of IA by the detection of Aspergillus antigen components [6]. Although the mortality rate for aspergillosis has been declining from 60 to 70% to around 40% [7], new laboratory methods that impart correct and rapid results are required for the diagnosis of this infection.

Procalcitonin (PCT) is processed in the thyroid gland (C cells) in normal individuals, and its level in healthy people is < 0.1 ng/ml [8]. PCT level is determined routinely in intensive care and surgical wards for the diagnosis of shock or respiratory distress syndrome, and also for early detection of postoperative infection diseases [9, 10]. The triggering receptor expressed on myeloid cells modulates the innate response by amplifying or dampening toll-like receptors [9]. It was identified as a regulator for innate and adaptive immune responses to infection [11, 12]. The extracellular domain of the triggering receptors expressed on myeloid can be found as soluble triggering receptors expressed on myeloid cells-1 (sTREM1) [9]. Nevertheless, the ability of sTREM-1 to diagnose infectious diseases remains controversial. As reported by Kollef et al., plasma sTREM-1 may be involved in lymphocytic regulation and differentiation during fungal infections [13]. According to recent studies, sTREM1 is elevated in plasma and bronchoalveolar lavage fluid from infected patients with bacterial or fungal pneumonia [14, 15]. Unlike bacterial infections, the value of PCT and sTREM-1 in detecting IA in immunocompromised patients is not clear. There are limited data on PCT, sTREM-1, C-reactive protein (CRP), lactate dehydrogenase (LDH), erythrocyte sedimentation rate (ESR) levels, and white blood cells (WBC) count in the diagnosis of aspergillosis [8, 10]. Therefore, this study aimed to investigate the roles of these biomarkers in the blood of immunocompromised patients with proven/probable IA, which can be helpful in antifungal stewardship programs.

## Methods

From June 2018 to June 2020, immunocompromised patients with suspected probable/proven IA were admitted to Shiraz University Hospitals, southern Iran enrolled in this study. Patients with signs and symptoms of infection without a clear-cut diagnosis were excluded. The diagnosis of infection was established in high-risk patients (immunocompromised) with clinical signs and symptoms of IA, and lab examination tests according to the European Organization for Research and Treatment of Cancer and the Mycoses Study Group (EORTC/MSG) [16]. Demographic characteristics including age, sex, background disease, clinical signs and symptoms, also WBC count, CRP, ESR, LDH values, site of infection, histopathological evidence, laboratory, and radiologic findings were extracted from patients’ files.

To avoid sample contamination, lab procedures were handled in biological safety cabinet class 1. Clinical samples like sputum, bronchoalveolar fluid, wound, tissue, and pleural effusion were examined by wet mount KOH smear and cultured in Sabouraud dextrose agar medium (Merck, Germany). The identification of *Aspergillus* spp. was achieved by macroscopic and microscopic evaluation of the isolates. As the sensitivity of cultures is limited, circulating DNA in the blood and other clinical samples were extracted and detected by real-time PCR [17]. The galactomannan test (PlateliaTM Ag assay; Bio-Rad, Germany) was performed in sera and bronchoalveolar samples of the patients according to the manufacturer’s instructions. Sera of patients were evaluated for PCT and sTREM-1 levels by ELISA Kits (Shanghaicrystal Day Biotech co. LTD, China). Eighteen immunocompromised patients with negative results of culture and molecular tests for any infections and galactomannan test were evaluated as control.

Data were analyzed using SPSS version 16. The data were not normal according to Kolmogorov-Smirnov and Shapiro-Wilk. Mann-Whitney test was used to compare variables and evaluate differences between groups. The sensitivity, specificity, odds ratio, and receiver operating characteristic (ROC) curves of the different assays were calculated using Prism 9.0 (Graph-Pad Software). Positive predictive values (PPV) and negative predictive values (NPV) were analyzed with MedCalc statistical software. The results were statistically significant at pv < 0.05.

## Results

One hundred ninety sera from 52 immunocompromised patients and volunteers were included in this study (18 immunocompromised volunteers without IA as control and 34 patients with probable and proven IA). Male-to-female ratios in patients were 22 (64.7%) and 12 (35.3%). The median age of participants was 39 years (range: 12–63 years), respectively. The hematologic disorder was the most predisposing factor (29/34, 85.3%) and respiratory system infection (lung and sinuses) was the most complicated organ (13/34, 38.2%) among the patients (Table 1). The patient’s clinical and radiological signs and symptoms were fever, cough, breathlessness, and pulmonary infiltrate. Both real-time PCR and GM tests were positive in two clinical samples of patients. Fungal cultures were positive in 26 patients, 21 *Aspergillus flavus* were isolated from sinuses tissue and bronchoalveolar lavage fluids, and five *Aspergillus fumigatus* from the central nervous system, and bronchoalveolar lavage fluids.

**Table 1:** Clinical data and outcome of patients with invasive aspergillosis in Shiraz, Iran

	Frequency	Percent
Sex		
Male	22	64.7
Female	12	35.3
Back ground		
Hematologic Disorder	29	85.3
Transplant	2	5.9
Combined Immunodeficiency	3	8.8
Site of infection		
Lung	11	32.3
Blood (FUO^⁕^)	10	29.4
Liver	6	17.7
Heart	1	2.9
Kidney	2	5.9
Sinuses	2	5.9
Central nervous system	2	5.9
Outcome		
Lived	22	64.7
Dead	12	35.3

*FUO* fever of unknown origin

**Fig. 1:**
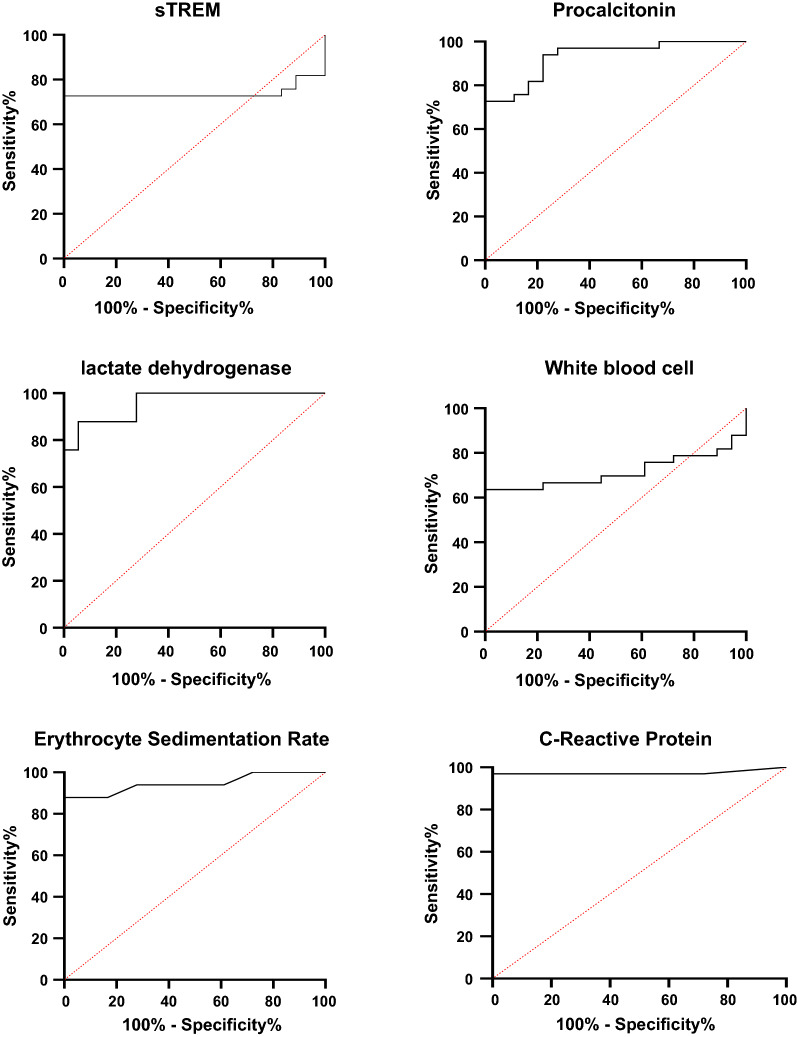
Receiver operating characteristics curves for evaluating the diagnostic role of biomarkers in patients with invasive aspergillosis

**Fig. 2:**
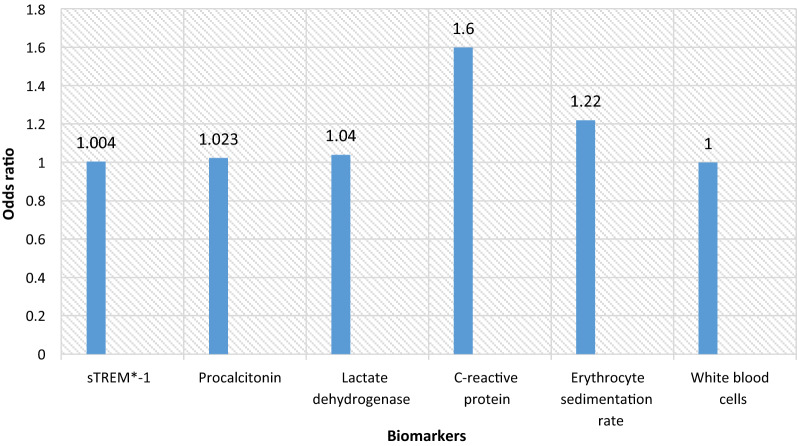
The odds ratio of biomarkers (1 = no effect of diagnosis, > 1 effect of diagnosis on patients suffering from invasive aspergillosis)

Receiver operating characteristics curves for evaluating the diagnostic role of biomarkers in patients suffering from IA was shown in Fig. 1. The AUC, cut-off value, sensitivity, specificity, PPV, and NPP of biomarkers measurement are shown in Table 2. The cut-off values > 190 pg/mL, > 260 pg/mL, > 193 U/L, > 8 mg/l, > 23 mm/h, and < 5200 cells/l in patients with IA were observed for sTREM-1, PCT, LDH, CRP, ESR, and WBC count, respectively. The AUC (95% confidence interval) for each biomarker were 0.97 (0.92 to 1) for CRP, 0.96 (0.92 to 1) for LDH, 0.95 (0.88 to1) for ESR, 0.93 (0.90 to 1) for PCT, 0.74 (0.59 to 0.88) for sTREM-1, and 0.72 (0.57 to 0.85) for WBC count. The odds ratios of CRP (1.60 with 95% CI: 1.04 to 2.46%) and ESR levels (1.22 with 95% CI: 1.03 to 1.45%) were observed the highest in the evaluated biomarkers (Fig. 2).

**Table 2:** The area under the curve (AUC), cut-off values, sensitivity, specificity, positive predictive value (PPV), and negative predictive value (NPP) for soluble triggering receptor expressed on myeloid cells-1 (sTREM-1), procalcitonin, lactate dehydrogenase, C-reactive protein, white blood cells count, and erythrocyte sedimentation rate in invasive aspergillosis cases

			P value < 0.001			
	sTREM*-1	Procalcitonin	Lactate dehydrogenase	C-reactive protein	White blood cells	Erythrocyte sedimentation rate
AUC	0.74	0.93	0.96	0.97	0.71	0.95
95% Confidence interval	0.59 to 0.88	0.86 to 0.99	0.91 to 1	0.92 to 1	0.57 to 0.85	0.88 to 1
Cut-off value	> 190pg/mL	> 260pg/mL	> 193U/L	> 5 mg/L	< 5200 cell/L	> 19 mm/h
Sensitivity	70.6%	71%	85.3%	94.1%	64.7%	85.3%
Specificity	100%	100%	94.1%	100%	100%	100%
PPV	100%	100%	96.7%	100%	100%	100%
NPV	63%	63%	76.2%	89.5%	58.6%	77.3%

The median (interquartile range, IQR) values of sTREM-1, PCT, LDH, WBC count, ESR, and CRP in patients were 1008.8 (79.2–1507) pg/mL, 357 (214.8-775.8) pg/mL, 474 (269–1371) U/L, 3050 (400–7800) cells/ml, 74 (41–97) mm/h, and 57 (26.5–132) mg/l, respectively.

A significant negative relationship was seen between WBC count and PCT level (pv = 0.05), and ESR value (pv = 0.002). While PCT or ESR values increased the WBC count decreased. Twenty-eight patients (28/34, 82.4%) received antifungal agents as prophylaxis or empiric therapy. There is significant differentiation between median of WBC count and the use of antifungal agents in patients (pv = 0.03). The WBC count was lower in patients (median 1800) who received antifungal agents than in patients not receiving ones (median 8100). Twelve patients (12/34, 35.3%) died. There were significant correlations between the patients’ outcomes and sTREM-1 (pv = 0.02) and WBC counts (pv = 0.005). The median sTREM-1 level in the dead patients was higher than in alive patients (1295 versus 291 pg/mL) while the WBC count was lower (400 versus 4100 cells/l).

## Discussion

Suitable treatment needs early diagnosis of IA. According to the literature, 7.7% of patients admitted to the intensive care unit and about 3% of hospital admissions are received systemic antifungals in the United States [18, 19]. Many of these about 30–50% are inappropriate [20, 21]. Over-prescription of antifungal agents can lead to drug interactions and toxicity in patients and resistance in fungi species [22].

In the present study, we evaluated the diagnostic significance of PCT, sTREM-1, ESR, CRP, LDH values, and WBC count in patients with IA. As revealed, pulmonary infections (lung and sinuses) were the most frequent complication among the patients. Invasive aspergillosis is predominantly present in the respiratory tract system and its dissemination from pulmonary sites to the brain, liver, kidney, skin, and gut was reported [23, 24]. Early diagnosis of IA in immunocompromised patients plays an important role in the patient’s outcome [25]. Yet, it is difficult because the result of blood culture may be rarely positive, the clinical signs and symptoms of IA are nonspecific, and isolation of etiologic agents from non-sterile samples like sputum may be due to colonization. Isolated species from sterile samples and the presence of fungal elements in tissues are the gold standards for the diagnosis of proven infection [14]. However, it is difficult to obtain sterile samples from patients with defective immune systems, and requires invasive procedures. *Aspergillus* antigen and antibody testing, detection of *β*-D-glucan as the pan-fungal antigen, *Aspergillus* lateral-flow device, and molecular-based assays are the other diagnostic methods. Using such methods in some regions is pending or very expensive. The use of the PCT level has been proposed as a new discriminative marker of infections [26].

Unfortunately, there are limited data in the literature about the role of the PCT level as a diagnostic marker for IA. The cut-off value for PCT > 260 pg/mL with a sensitivity of 80.8% and specificity of 100% was reported in pediatric patients suffering from IA [27]. In Robinson et al., after 3 days, a PCT level > 500 pg/mL occurred in 81% (17/21) of the patients with invasive fungal diseases [28]. The mean PCT concentration in 86 healthy individuals was reported at 15.8 pg/mL by Carcamo and coworkers [29]. Also, in healthy people, PCT concentration was reported below 50 pg/mL (0.05 ng/ml] [29] and 100 pg/mL (< 0.1 ng/ml) [8]. Ortega and co-workers reported: in five patients with more than 5 days of persistent fever, the cutoff level was ≥ 3 ng/ml for the diagnosis of IA [9]. Marková and co-workers reported in hematological patients with IA, PCT elevation was minimal or negative (p < 0.01) [30]. In the present study, the optimum cut-off value in the diagnosis of IA was > 260 pg/mL for PCT. Our data like other studies [8, 28, 29] showed a low elevation of PCT in patients with IA. The PCT level in patients with IA is lower because *Aspergillus* species have a tendency for local infection and isolation of this organism from the blood is rare [25].

The ability of sTREM-1 in the diagnosis of IA remains controversial. According to the literature, during infection, sTREM-1 could be released into the body fluids and could be used as the diagnostic biomarker for bacteremia [31]. For the early detection of infectious diseases, sTREM-1 is a feasible, sensitive, and specific biomarker [32]. In the differentiation of sepsis, sTREM-1 cut-off value ≥ 133 pg/mL yielded a sensitivity of 71.1% and specificity of 73.3% [33]. Also, the value of 300 pg/mL was determined to be the optimal cut-off value for sTREM-1 as a diagnostic biomarker of sepsis [34]. The value of this marker in IA is not clear. Buckland et al. reported circulating sTREM-1 levels in experimental fungal asthma peaked on day 30 in the mice model [35]. The cut off value for sTREM1 > 190 pg/mL with sensitivity 71.2%, specificity 100%, PPV 100% and NPPV 46.2% was reported in infected pediatric patients with *Aspergillus* species [27]. In the literature, we could not find a study that included the cut-off value of sTREM-1 in patients suffering from IA.

High circulating CRP levels may be an initiation of the diagnosis of IA. In patients with a poor response to antifungal treatment, high CRP levels may be early predictors of adverse outcomes [36]. A significant increase in CRP levels was reported in IA, chronic pulmonary aspergillosis, and aspergilloma [37, 38]. In 22 patients with IA, CRP peak levels were reported to rang from 5 to 384 mg/l, and a median of 58 mg/l [39]. In the present study, the AUC of CRP was higher than that of other biomarkers. The cut-off value for CRP > 7 mg/l with a sensitivity of 90.4% and specificity of 100% was reported were reported in patients with IA [27]. Lactate dehydrogenase is an enzyme required for energy production for human cells and is released into the bloodstream during cell damage. In those with invasive pulmonary aspergillosis, a very high LDH level was reported (1977 U/L) [40]. Jhun et al. reported that 17 (24%) patients with chronic pulmonary aspergillosis had a WBC count greater than 10,000 cells/µl with the median ESR value being 78 mm/h [38]. The WBC count in Chinese patients with chronic cavitary pulmonary aspergillosis, semi-invasive aspergillosis, and simple aspergilloma reported 6800, 12,900, and 6500 cells/ml, respectively (pv = 0.0001) [37]. The ESR value in patients with invasive pulmonary aspergillosis was reported 46 mm/h (n < 20) [40]. In patients with chronic pulmonary aspergillosis, significant differences in WBC count, ESR, and CRP value were reported (all P < 0.05) pre and post-diagnosis [37]. In the study by Rajalingham and associates, in patients with chronic necrotizing pulmonary aspergillosis, WBC count was in the normal range, and a rise in CRP (3.24 mmol/L) and ESR (66 mm/hour) were seen [41]. In this study like other studies, the performance of ESR and CRP with higher odds ratios were found to be more significant than the performance of WBC count in patients.

In the current study, only a significant differentiation between the median of WBC count and the use of antifungal agents in patients was seen and there was no differentiation between the median of other biomarkers and the use of antifungal agents. Because, despite the use of antifungal agents as prophylaxis, the patients had an invasive *Aspergillus* infection. Assicot and co-workers in patients with bacterial infections reported that “the serum levels of PCT would decrease following administration of appropriate antibiotic therapies” [42]. Ling Cheng and co-workers by ROC curve analysis reported that PCT concentrations of more than 1.31 ng/mL increased the incidence of a voriconazole trough level higher than 5 µg/mL [43]. The ESR, CRP, and sTREM-1 values in pediatric patients with IA were increased in patients using antifungal agents [27]. A decrease in biomarkers in patients using antifungal agents presents the effectiveness of treatment [44]. If antifungal therapy was not effective, the biomarker levels did not decrease and evaluation of these factors can help antifungal stewardship programs.

There was some limitation in the present study. The sample size was small and we could not have evaluated these biomarkers on the different days of infection diagnosis. Also, most background patients were hematologic disorders, with many using antibiotics for treatment or prophylaxis of different infections. Additional evidence from large-scale multi-center studies with a high number of patients is required to support the sensitivity, specificity, and prognostic role of inflammatory factors in the diagnosis of IA.

## Conclusion

According to our data, the cut-off values for biomarkers in the diagnosis of IA were reported. Since biomarkers are unspecific inflammatory markers, evaluating of these along with patients’ clinical and radiologic signs and symptoms can lead to proper antifungal therapy and decreased side effects, antifungal resistance, and cost. we found that CRP, LDH, ESR, and PCT had higher performances than sTREM-1 and WBC count for the diagnosis of IA. In patients with clinical signs and symptoms, low or normal WBC count, and high-level values of PCT, sTREM-1, CRP, LDH, and ESR, the possibility of IA and the use of antifungal agents should be considered. In this study, dead patients presented significantly higher sTREM-1 and lower WBC count.

## Data Availability

The datasets used and/or analyzed during the current study are available from the corresponding author upon reasonable request.

## Abbreviations

IA: invasive aspergillosis
PCT: procalcitonin
sTREM-1: soluble triggering receptor expressed on myeloid cells
CRP: C-reactive protein
LDH: lactate dehydrogenase
ESR: erythrocyte sedimentation rate
EORTC/MSG: European Organization for Research and Treatment of Cancer and the Mycoses Study Group
ROC: receiver operating characteristic
AUC: area Under the Curve
PPV: positive predictive value
NPP: negative predictive value
